# Tracking Control for a Lower Extremity Exoskeleton Based on Adaptive Dynamic Programing

**DOI:** 10.3390/biomimetics8040353

**Published:** 2023-08-09

**Authors:** Qiying Su, Zhongcai Pei, Zhiyong Tang

**Affiliations:** School of Automation Science and Electrical Engineering, Beihang University, 37 Xueyuan Road, Haidian District, Beijing 100191, China; suqiying@buaa.edu.cn (Q.S.); peizc@buaa.edu.cn (Z.P.)

**Keywords:** lower extremity exoskeleton, tracking control, adaptive dynamic programming, value iteration

## Abstract

The utilization of lower extremity exoskeletons has witnessed a growing presence across diverse domains such as the military, medical treatment, and rehabilitation. This paper introduces a novel design of a lower extremity exoskeleton specifically tailored for individuals engaged in heavy object carrying tasks. The exoskeleton incorporates an impressive 12 degrees of freedom (DOF), with four of them being effectively controlled through hydraulic cylinders. To achieve optimal control of this intricate lower extremity exoskeleton system, the authors propose an adaptive dynamic programming (ADP) algorithm. Several crucial components are established to implement this control scheme. These include the formulation of the state equation for the lower extremity exoskeleton system, which is well-suited for the ADP algorithm. Additionally, a corresponding performance index function based on the tracking error is devised, along with the game algebraic Riccati equation. By employing the value iteration ADP scheme, the lower extremity exoskeleton demonstrates highly effective tracking control. This research not only highlights the potential of the proposed control approach but also showcases its ability to enhance the overall performance and functionality of lower extremity exoskeletons, particularly in scenarios involving heavy object carrying. Overall, this study contributes to the advancement of lower extremity exoskeleton technology and offers valuable insights into the application of ADP algorithms for achieving precise and efficient control in demanding tasks.

## 1. Introduction

The control technology of wearable exoskeletons has emerged as a prominent area of research worldwide, driven by its extensive applications in medical treatment, rehabilitation, and load-bearing tasks. In particular, load-bearing exoskeletons, with the aid of energy supply, have proven effective in enhancing human intelligence and adaptability [1]. The control process of exoskeletons is typically divided into two levels: upper level control and lower level control. The upper level control aims to capture the wearer’s motion intention and generate position or force signals as input for actuators such as hydraulic cylinders, motors, or pneumatic muscles. This process, known as trajectory generation, plays a crucial role in guiding the exoskeleton’s movements. On the other hand, the lower level control ensures that the exoskeleton accurately follows the trajectory generated by the upper level control [2]. Given the pivotal role of upper level control in the overall exoskeleton control process, researchers have increasingly focused on developing highly adaptive control algorithms for exoskeletons. These algorithms aim to improve the exoskeleton’s ability to adapt to various wearer motions and optimize its performance in real-world scenarios. By advancing the field of upper level control, researchers aim to enhance the functionality, versatility, and overall user experience of exoskeleton systems. This research direction holds significant promise for the future development of wearable exoskeleton technology, enabling its wider application and impact in diverse fields. Overall, the exploration of adaptive control algorithms for exoskeletons represents a critical area of research, with the potential to revolutionize the capabilities and effectiveness of these systems in assisting individuals in their daily activities.

The Sensitivity Amplification Control (SAC) method, proposed by the University of California at Berkeley, aims to minimize the interactions between humans and machines while enhancing the exoskeleton’s ability to effectively track human movement trajectories [3]. Klong Luang et al. introduced the zero-moment point (ZMP) scheme to address balance issues in rehabilitation exoskeletons, which is also utilized in biped robots. However, this method is limited to achieving small steps [4]. Researchers have explored a hierarchical control scheme for exoskeletons to facilitate cooperation with humans. They developed an adaptive neural network controller based on admittance control and Gaussian mixture models [5]. R. M. Andrade et al. designed a six DOF exoskeleton, which can work in three different modes, based on the research into how the mechanical design of the robot can interfere with the user’s gait pattern and proposed an impedance controller for it [6,7]. Additionally, a human-in-loop control method was proposed for a unilateral exoskeleton system used in gait rehabilitation for hemiplegic patients. Real-time follower algorithms were employed to assist the patient’s movement [8]. Another control strategy based on internal perception was investigated, utilizing feedforward compensation and limit learning machines to improve the force tracking control and response speed to human motion intentions [9]. Furthermore, a robust controller was designed for a three DOF hydraulic leg exoskeleton, enabling accurate tracking of human motion [10]. Shiqian Wang designed a new type of exoskeleton called MINDWALKER actuated by SEA and proposed a novel step-width adaptation algorithm to stabilize the lateral balance [11]. These research efforts highlight various innovative control approaches for exoskeleton systems, addressing crucial aspects such as tracking accuracy, balance, cooperation with humans, and force control. By advancing these control methods, researchers aim to enhance the safety, effectiveness, and user experience of exoskeleton technology in applications such as rehabilitation, load-bearing tasks, and human assistance.

In recent years, the ADP (Approximate Dynamic Programming) algorithm has gained significant attention in academia and has been rapidly adopted in industry [12,13,14,15,16,17]. The remarkable control performance of the ADP algorithm in traditional control applications has demonstrated its capability to handle complex control tasks, attracting numerous researchers to study its theories and applications. Compared to traditional controllers that rely on the system’s input–output difference to calculate the control strategy, intelligent controllers employed in the ADP algorithm utilize system states to determine the control strategy. While the classical PID controller design is based on the system’s mechanism, the ADP algorithm requires a training model built through monitoring signals. Depending on the iteration rules, the ADP algorithm can be categorized into policy iteration [18,19,20,21,22] and value iteration [23,24,25,26]. Researchers have explored the application of the ADP control algorithm in exoskeleton controllers, achieving favorable control effects [27,28,29,30,31]. For example, an ADP algorithm was developed to address the inherent nonlinearity and parameter uncertainty of a robot system [27]. Motion sequence planning and motion adaptive coupling algorithms based on dynamic motion primitives were studied, and a trajectory learning scheme using reinforcement learning was proposed for a walking exoskeleton robot to assist human walking [28]. A novel control strategy based on learning was investigated for assisting hemiplegic patients in walking with an exoskeleton, employing an iterative ADP algorithm to achieve improved tracking control performance [29]. An approximate dynamic programming method was studied to automatically adjust the parameters of an exoskeleton knee prosthesis, catering to the individual needs of users and enabling online learning control [30]. An interactive learning method based on actor–critic was also applied to an exoskeleton system with a continuous high-dimensional observation space [31]. However, there is limited research on utilizing the ADP algorithm to design lower extremity exoskeleton controllers in the presence of disturbances. This paper proposes a new motion tracking controller based on the ADP algorithm, aiming to achieve optimal control of lower extremity exoskeletons. By leveraging the capabilities of the ADP algorithm, this controller can effectively handle disturbances and enhance the performance of lower extremity exoskeletons in motion tracking tasks. Overall, the application of the ADP algorithm in exoskeleton control represents a promising research direction, offering opportunities to improve the adaptability, robustness, and control performance of exoskeleton systems, particularly in the context of lower extremity exoskeletons.

This article introduces the design of an innovative exoskeleton robot and suggests a hydraulic drive system that utilizes the robot’s structure. The exoskeleton is equipped with a limited number of sensors, resulting in reduced costs. Moreover, a dedicated ADP control algorithm is devised for this robot. The algorithm demonstrates exceptional learning capabilities and precise control, effectively resolving the problem of ensuring accurate position tracking for the wearer within the exoskeleton system.

## 2. Design and Modeling of the Exoskeleton

### 2.1. Mechanical Design

The lower limb exoskeleton was designed as a wearable robot, taking into consideration the relevant parameters of the human lower limb to ensure optimal comfort for the operator. Therefore, the structural design of the lower limb exoskeleton adhered to the principles of ergonomics.

**Figure 1:**
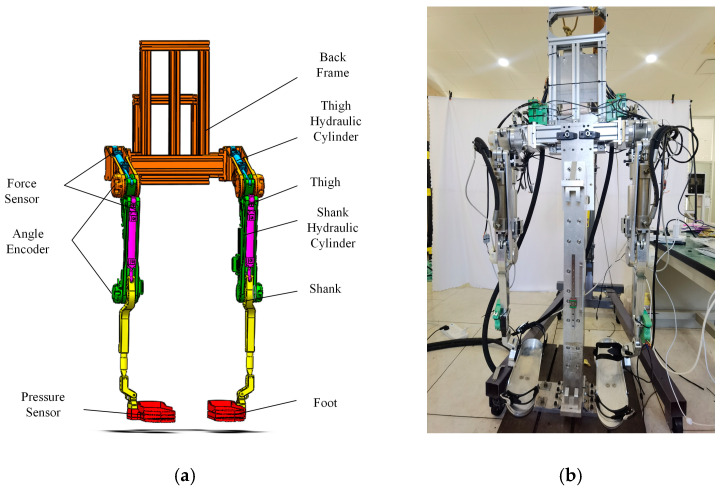
Structure of the exoskeleton. (**a**) Three-dimensional model of the exoskeleton. (**b**) The exoskeleton experimental platform.

The mechanical structure of the lower extremity exoskeleton is depicted in Figure 1a showcases the 3D model of the exoskeleton, while Figure 1b presents the physical experiment platform. These visual representations provide a clear understanding of the design and serve as a basis for further analysis and evaluation.

**Figure 2:**
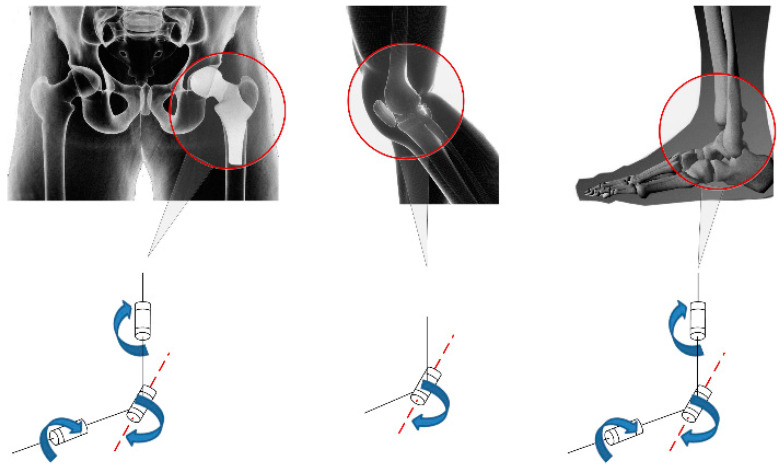
Simplified diagram of lower limb joints.

The DOFs of the lower extremity exoskeleton were designed according to the simplified DOFs of a human being, as shown in Figure 2.

**Figure 3:**
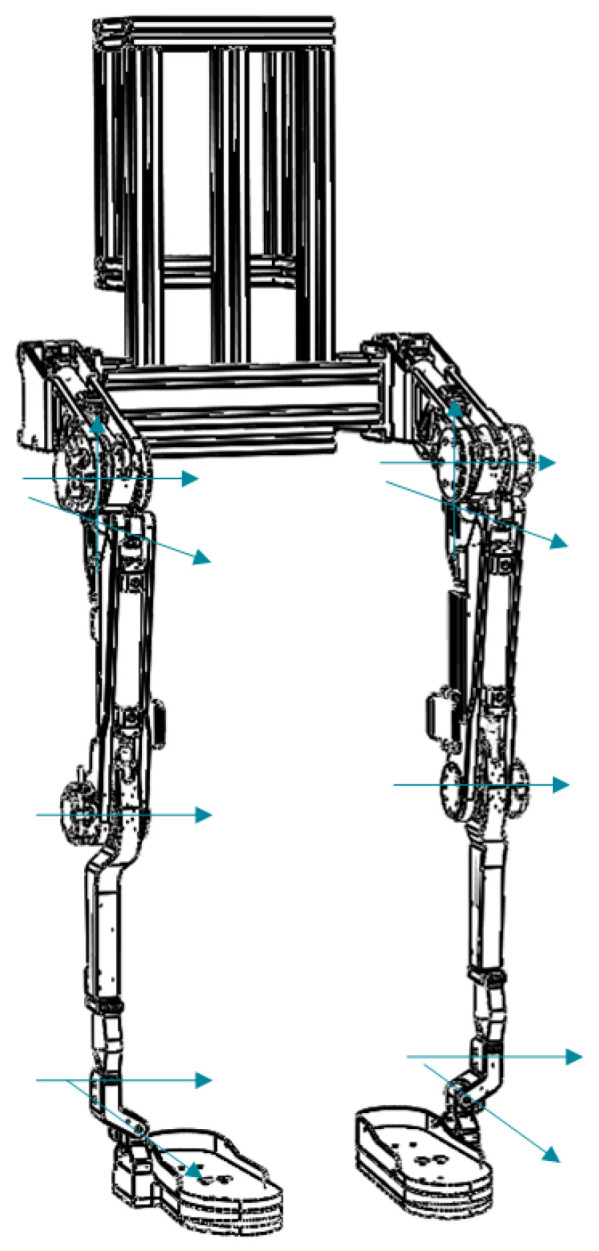
Joints of the exoskeleton.

The exoskeleton has a total of 12 DOFs, of which each hip joint has 3 rotation DOFs, each knee joint has 1 rotation DOF and each ankle joint has 2 rotation DOFs. The DOFs of the exoskeleton are shown in Figure 3.

**Figure 4:**
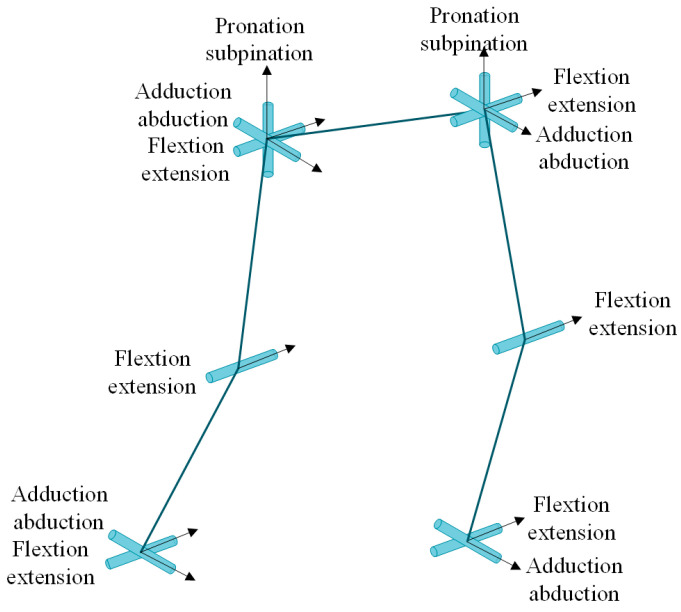
Joint DOFs of the exoskeleton.

After simplifying the exoskeleton mechanism to a linkage mechanism, the degrees of freedom were labeled on a diagram, as shown in Figure 4.

Due to the significant average power between the knee and hip joints during the human body’s forward movement and the torque mainly distributed in the forward direction, four of the twelve DOFs, including the flexion/extension DOFs of the hip joints and flexion/extension DOFs of the two knee joints, were actuated.

The exoskeleton was designed to operate in low-speed, high-load, and high-precision scenarios, particularly to assist pilots in carrying heavy objects. Therefore, the exoskeleton joints require a significant driving force. Taking advantage of the hydraulic drive’s characteristics such as the high-power density, compact size, light weight, stable low-speed performance, and precise control, this paper proposes the use of hydraulic cylinders as actuators in the exoskeleton design.

Considering that the joint motion range and average power of the knee and hip joints are much greater than those of the ankle joint during walking, and the primary joint movement is predominantly in the forward direction, the exoskeleton was equipped with hydraulic cylinders only at the knee and hip joints. This includes the flexion/extension degrees of freedom of the hip joints and the flexion/extension degrees of freedom of the two knee joints, which were actively actuated.

As the exoskeleton serves as a universal wearable device for pilots of varying heights and leg lengths, the thigh and calf sections of the exoskeleton were designed with an adjustable range. This feature enables the exoskeleton to accommodate pilots with heights ranging from 1.7 m to 1.85 m, thereby enhancing comfort and adaptability.

**Figure 5:**
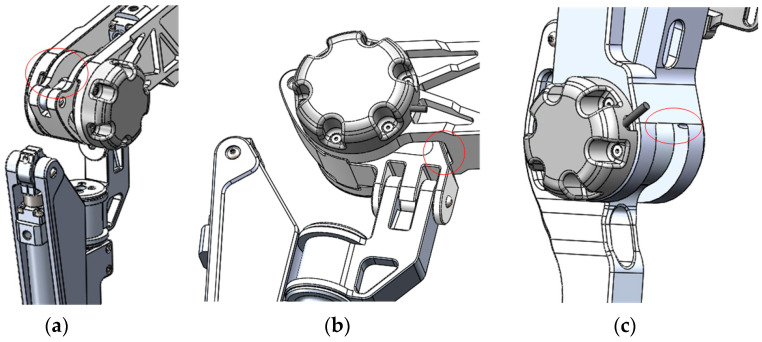
Limits of the exoskeleton. (**a**) Limit of the hip joint extension. (**b**) Limit of the hip joint flexion. (**c**) Limit of the hip joint extension.

To prioritize pilot safety, all the actuated joints of the exoskeleton were equipped with mechanical limits. These limits ensure that the joint motion range of the exoskeleton remains within the safe range of human joint movement during travel. By incorporating these safety measures, the exoskeleton provides a secure and controlled environment for pilots while using the device. The mechanical limits are shown in Figure 5.

The extension and flexion of the hip joint is shown in Figure 5a,b, and the flection of the knee joint is shown in Figure 5c; the extension of the knee joint limit is achieved by the hydraulic cylinder, and when the piston rod of the hydraulic cylinder is fully retracted, the knee joint angle is at its minimum.

The design specifications are shown in Table 1.

After the design of the structure, the strength of the structure was vitrificated by simulation; according to the CGA data [32,33] and research on Human Biomechanics data [33,34,35], the strength of this structure was reliable.

### 2.2. Hydraulic System

The lower limb exoskeleton designed in this research is mainly used for load-bearing and assisted walking. It utilizes hydraulic drive technology, which has advantages such as high power density, fast response speed, and strong load-bearing capacity. The hip and knee joints of the exoskeleton are driven by hydraulic cylinders to amplify human lower limb strength and achieve the goal of assisted walking.

**Figure 6:**
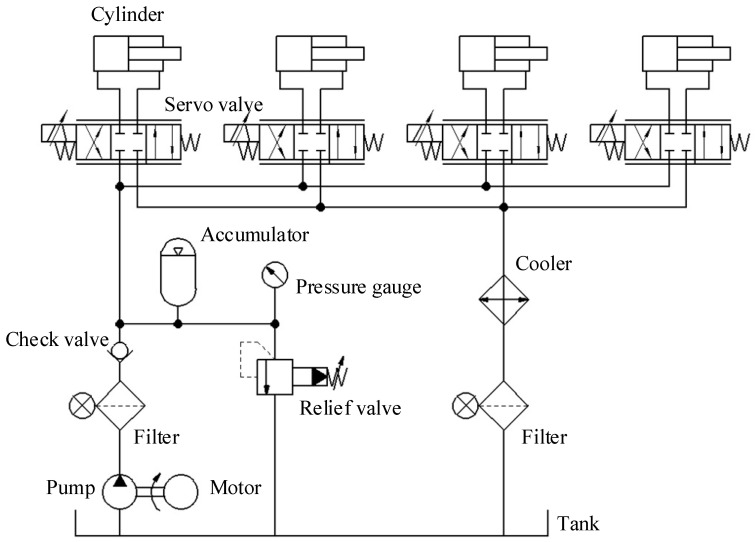
Schematic diagram of lower limb Exoskeleton hydraulic control system.

The principal diagram of the lower limb exoskeleton hydraulic control system is shown in Figure 6. Four hydraulic cylinders are independently controlled by four electro-hydraulic servo valves, which control the rotation of the hip and knee joints of the exoskeleton’s two legs, respectively. The hydraulic system adopted a constant pressure oil source, with the system operating pressure regulated by an overflow valve. An accumulator was installed in the system circuit as an auxiliary power source, working together with the hydraulic pump to provide the required flow and pressure for system movement.

**Figure 7:**
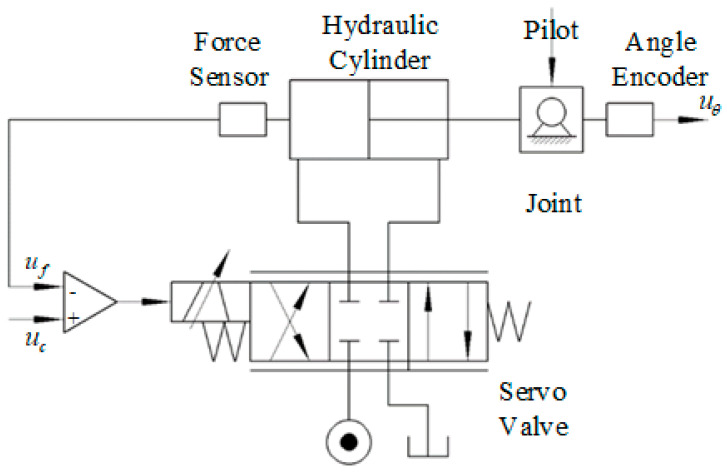
Principle diagram of the lower limb exoskeleton hydraulic control system.

The control structure of the exoskeleton hydraulic drive system is shown in Figure 7. The hydraulic valve-controlled cylinder drive method was adopted in the exoskeleton hydraulic drive system. When a person wears the exoskeleton and walks, the input signal of the valve-controlled cylinder force control system is generated through human–machine interaction. The output force of the valve-controlled cylinder actuator controls the rotation of the exoskeleton joints. Tension and compression force sensors are used to detect the output force of the hydraulic cylinder, forming a feedback loop to achieve the followup control of the driving force for the hip and knee joints.

### 2.3. Sensors

Accurate exoskeleton position and force information play a crucial role in the followup control of exoskeleton robots. In the exoskeleton robot designed in this research, the required information included the joint driving force, the joint angle position, and the plantar pressure.

**Figure 8:**
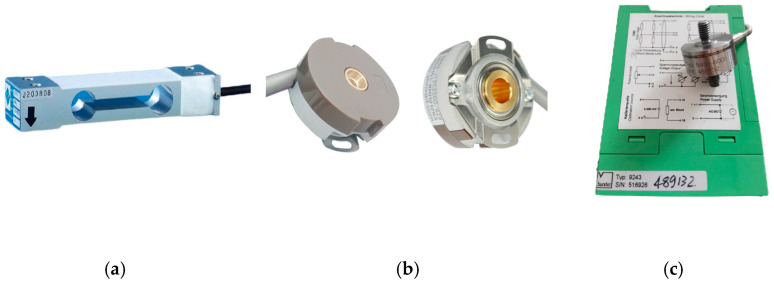
Sensors of the exoskeleton. (**a**) Pressure sensor. (**b**) Angle encoder. (**c**) Precision load cell.

During the entire exoskeleton walking process, each leg is divided into two phases, which are the swing phase and the support phase. The two phases correspond to different model parameters, and sensors need to be used to determine the phase of each leg of the exoskeleton. The most intuitive difference between the swing phase and the support phase is whether there is contact force between the foot and the ground. Therefore, two pressure sensors were used to determine the phase of the exoskeleton. The plantar pressure sensor used is shown in Figure 8a. The sensor had a range of 0–250 kg and an error of 0.2%, and its shape was regular, suitable for installation, and met the maximum plantar pressure measurement requirements of normal wearers during the walking process.

Four incremental rotary encoders were used to measure the angle positions of the four actuated joints, providing input signals for the upper control. The KN-40 hollow shaft rotary encoder and its installation position are shown in Figure 8b. This type of angle encoder has the characteristics of a small volume, light weight, and high accuracy, with a thickness of only 20 mm, suitable for the joint position of exoskeleton robots.

The hydraulic cylinder tension pressure sensor and its installation position are shown in Figure 8c. The 8431–6005 tension pressure sensor from Bosch Company in Germany was selected, with a nonlinearity of 0.2% and a rated tension pressure of −50 kN–50 kN. Due to the output voltage of this sensor itself, an amplifier from the same company was selected for power amplification.

## 3. Modeling

The design purpose of the lower limb exoskeleton is to assist the wearer in bearing weight. While bearing weight, the exoskeleton needs to track the wearer’s walking trajectory to avoid additional loads on the wearer’s joints. The ADP algorithm designed in this work was a model-based control algorithm. Therefore, before designing the control algorithm, dynamic modeling of the lower limb exoskeleton was performed.

**Figure 9:**
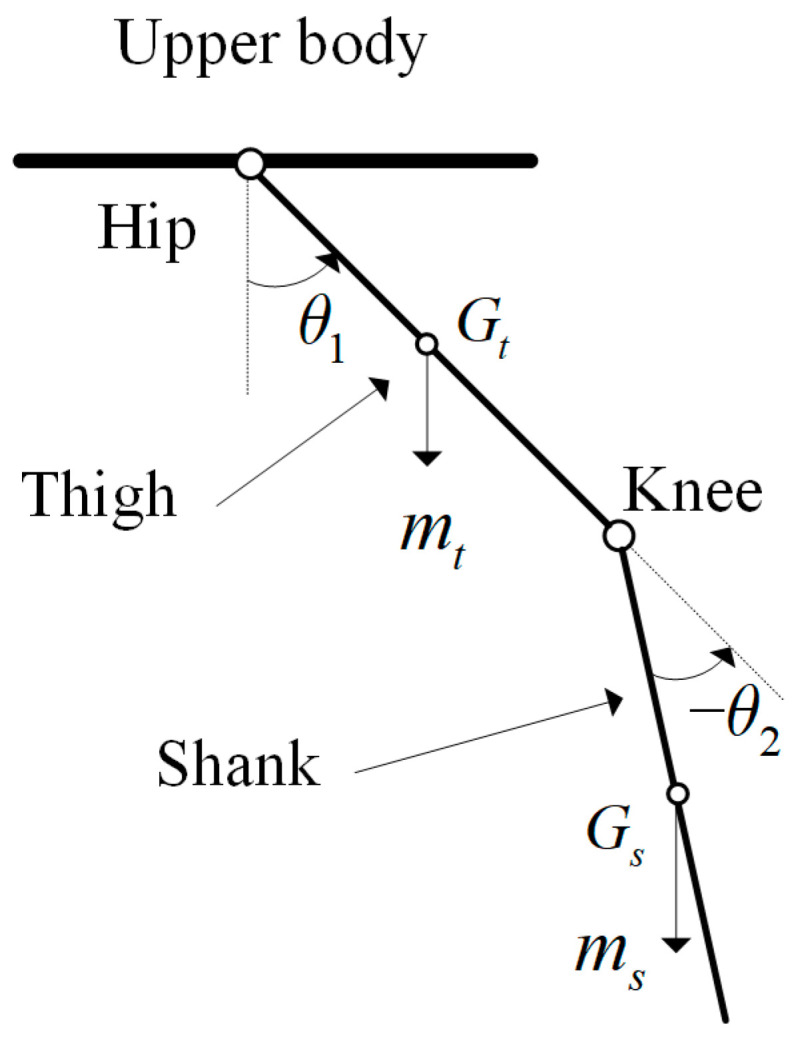
Simplified two-link model of the swing phase.

In this work, the two legs of the exoskeleton were controlled separately, and only the knee joint and the hip joint were actuated by the hydraulic cylinder; so, the ankle joint was ignored while modeling. Therefore, each leg of the exoskeleton could be seen as a two-link system. The simplified connecting rod structure is shown in Figure 9.

The generated coordinates are as follows:

- $\theta_{1}$: the angle of thigh with hip;
- $\theta_{2}$: the angle of shank with thigh;
- $G_{t}$: the angle of shank with thigh;
- $G_{s}$: shank’s center of gravity;
- $m_{t}$: thigh’s quality;
- $m_{s}$: shank’s quality.

Generally speaking, the Lagrange method is a commonly used dynamic modeling method for link systems. For the exoskeleton single-leg two-link mechanisms, the dynamic model was established. The general two-ink dynamic model is as Equation (1):

(1) $$\tau =M(\theta )\ddot{\theta }+C(\theta ,\dot{\theta })\dot{\theta }+G(\theta ).$$

In Equation (1), $\theta ={\left[\begin{array}{cc} \theta_{1} & \theta_{2} \end{array}\right]}^{T}$, where $\theta_{h}$ is the angular position of hip joint, and $\theta_{k}$ is the angular position of knee joint; $\tau ={\left[\begin{array}{cc} \tau_{1} & \tau_{2} \end{array}\right]}^{T}$, where $\tau_{1}$ is the moment of the hip joint, and $\tau_{2}$ is the moment of the knee joint; M(θ) is the inertia matrix; $C(\theta ,\dot{\theta })$ is called centripetal and Coriolis matrix; and G(θ) is the matrix of the gravitational.

The specific expression of matrices M(θ), $C(\theta ,\dot{\theta })$, and G(θ) can be written as:

$$M(\theta )=\left[\begin{array}{cc} M_{11} & M_{12}\\ M_{21} & M_{22} \end{array}\right],\;C(\theta ,\dot{\theta })=\left[\begin{array}{cc} C_{11} & C_{12}\\ C_{21} & C_{22} \end{array}\right],\;G(\theta )==\left[\begin{array}{c} G_{1}\\ G_{2} \end{array}\right],$$

where $m_{11}=m_{t}{\left(L_{Gt}-L_{t}\right)}^{2}+I_{t}+m_{s}L_{t}{}^{2}+m_{s}{\left(L_{s}-L_{Gs}\right)}^{2}+I_{s}+2m_{s}L_{t}(L_{s}-L_{Gs})\cos\theta_{2}$,

$$m_{12}=m_{s}{\left(L_{s}-L_{Gs}\right)}^{2}+I_{s}+m_{s}L_{t}(L_{s}-L_{Gs})\cos\theta_{2},$$

$$m_{21}=m_{s}{\left(L_{s}-L_{Gs}\right)}^{2}+I_{s}+m_{s}L_{t}(L_{s}-L_{Gs})\cos\theta_{2},$$

$$m_{22}=m_{s}{\left(L_{s}-L_{Gs}\right)}^{2}+I_{s},$$

$$c_{11}=-2m_{s}L_{t}(L_{s}-L_{G}s)sin(\theta_{2}){\dot{\theta }}_{2},$$

$$c_{12}=-m_{s}L_{t}(L_{s}-L_{Gs})sin(\theta_{2}){\dot{\theta }}_{2},$$

$$c_{21}=m_{s}L_{t}(L_{s}-L_{Gs})sin(\theta_{2}){\dot{\theta }}_{1},$$

$$c_{22}=0,$$

$$g_{1}=[m_{t}g(L_{t}-L_{Gt})+m_{s}gL_{t}]\sin q_{1}+m_{s}g(L_{s}-L_{G}s)\sin(\theta_{1}+\theta_{2}),$$

$$g_{2}=m_{s}g(L_{s}-L_{Gs})\sin(\theta_{1}+\theta_{2}),$$

where m is the mass of each pole, L represents the length of each pole, I is the moment of inertia of each pole around the center of mass, $L_{G}$ is the distance from the center of mass to the axis of rotation of the pole, the subscript t is the thigh, and the subscript s is the shank.

In this paper, the state equation of the exoskeleton can be defined as

(2) $$\dot{x}=f(x)+g(x)u,$$

where $x={\left[\begin{array}{cccc} x_{1} & x_{2} & x_{3} & x_{4} \end{array}\right]}^{T}={\left[\begin{array}{cccc} \theta_{1} & \dot{\theta_{1}} & \theta_{2} & \dot{\theta_{2}} \end{array}\right]}^{T}$, $f(x)={\left[\begin{array}{cccc} f_{1}(x) & f_{2}(x) & f_{3}(x) & f_{4}(x) \end{array}\right]}^{T}$ and $g(x)=[\begin{array}{cc} \begin{array}{cc} g_{11}(x) & g_{12}(x) \end{array};g_{21}(x) & g_{22}(x) \end{array};\begin{array}{cc} g_{31}(x) & g_{32}(x) \end{array};\begin{array}{cc} g_{41}(x) & g_{42}(x) \end{array}]$, in which $f_{1}(x)=f_{3}(x)=g_{11}(x)=g_{12}(x)=g_{31}(x)=g_{32}(x)=0$, $f_{2}(x)=\frac{1}{S}[M_{12}(C_{2}+G_{2})-M_{22}(C_{1}+G_{1})]$, $f_{4}(x)=-\frac{1}{S}[M_{11}(C_{2}+G_{2})-M_{12}(C_{1}+G_{1})]$, $g_{21}(x)=\frac{M_{22}}{S}$, $g_{22}(x)=-\frac{M_{12}}{S}$, $g_{41}(x)=-\frac{M_{21}}{S}$, and $g_{42}(x)=\frac{M_{11}}{S}$.

## 4. Game Algebraic Riccati Equation

The linearized description of the exoskeleton model (2) can be written as:

(3) $$\dot{x}=Ax+B(u+d_{1})$$

where $x\in R^{m\times 1}$, $u\in \;R^{p\times 1}$, and $d\in R^{p\times 1}$ represent the system state, the measured output, the control input, and the disturbances, respectively. $A\in R^{m\times m}$, $B\in R^{m\times p}$, are known constant system matrixes.

The corresponding reference state equation is:

(4) $$\dot{r}=Ar+B(u+d_{2}).$$

Based on (3) and (4), the tracking error has the following form:

(5) $$\dot{\tilde{x}}=A\tilde{x}+B(u+\omega ),$$

where $\dot{\tilde{x}}=A\tilde{x}+B(u+\omega )$, $\dot{\tilde{x}}=A\tilde{x}+B(u+\omega )$, and the control policy is assumed to have the form $\dot{\tilde{x}}=A\tilde{x}+B(u+\omega )$ and $\dot{\tilde{x}}=A\tilde{x}+B(u+\omega )$.

The corresponding performance index function of the lower extremity exoskeleton is defined as:

(6) $$\begin{array}{ccc} J(u,\omega ) & = & \int_{0}^{\infty }Ud\tau \\ & = & \int_{0}^{\infty }({\tilde{x}}^{T}Q\tilde{x}+u^{T}Ru-\gamma^{2}\omega^{T}\omega )d\tau \end{array}.$$

With R and Q≥0, U represents the utility function, and

$$\int_{0}^{\infty }u^{T}Rud\tau \leq \int_{0}^{\infty }\gamma^{2}\omega^{T}\omega d\tau .$$

Assume that the pair (A, B) is stabilizable and (A,$\sqrt{Q}$) is detectable. Then, there exists a control law to make the controlled system (1) stable [36].

For the optimal control problem of an exoskeleton, $u^{*}$ and $\omega^{*}$ satisfy the following relationship:

(7) $$J^{*}(u^{*},\omega^{*})=\underset{u}{\min}\underset{\omega }{\max}J(u,\omega )=\underset{\omega }{\max}\underset{u}{\min}J(u,\omega ).$$

Based on (6), we define a performance index function:

(8) $$V(u,\omega )=\int_{t}^{\infty }\left({\tilde{x}}^{T}Q\tilde{x}+u^{T}Ru-\gamma^{2}\omega^{T}\omega \right)d\tau .$$

According to the Bellman principle, the Hamiltonian function is given by:

(9) $$H(\tilde{x},\nabla V,u,\omega )=U(\tilde{x},u,\omega )+\nabla V^{T}(A\tilde{x}+Bu+B\omega ).$$

Here, ∇V is the partial derivative of V.

Then, the Hamilton–Jacobi–Isaacs (HJI) equation is given by:

(10) $$H(\tilde{x},\nabla V^{*},u^{*},\omega^{*})={\tilde{x}}^{T}Q\tilde{x}+\nabla V^{*}T(A\tilde{x}+Bu+B\omega ).$$

Obviously this is a zero-sum game problem, which can obtain P with the help of the game algebraic Riccati equation.

(11) $$A^{T}P+PA+Q-PBR^{-1}B^{T}P+\gamma^{-2}PBB^{T}P=0$$

The corresponding u and ω have the following form:

(12) $$u=-K_{1}\tilde{x}=-R^{-1}B^{T}P\tilde{x},$$

(13) $$\omega =K_{2}\tilde{x}=\gamma^{-2}B^{T}P\tilde{x}.$$

The goal of the lower extremity exoskeleton control is to obtain the optimal control $u^{*}$, so that the controlled system (3) tracks the desired reference trajectory (4) in an optimal way by minimizing the predefined performance indicators (6).

## 5. ADP Algorithm for the Control of the Swing Phase

The optimal control problem is to minimize the energy control $\int_{0}^{\infty }u^{T}Rud\tau$, in the case of disturbance.

According to the performance index function (6), the following function V(x,e) can be defined:

(14) $$J(u,\omega )=\int_{0}^{\infty }e^{\delta \tau }(u^{T}Ru-\gamma^{2}\omega^{T}\omega )d\tau .$$

Then, according to (11)–(13), the matrix P satisfies:

(15) $${\left(A+\frac{\delta I}{2}\right)}^{T}P+P(A+\frac{\delta I}{2})-PBR^{-1}B^{T}P+\gamma^{-2}PBB^{T}P=0.$$

Then, (12) and (13) have the following form:

(16) $$u^{*}=-K_{1}^{*}\tilde{x}=-R^{-1}B^{T}P^{*}\tilde{x},$$

(17) $$\omega^{*}=K_{2}^{*}\tilde{x}=\gamma^{-2}B^{T}P^{*}\tilde{x}.$$

Note that (15) can be rewritten as:

(18) $$A-BK_{1}+BK_{2}=-P^{-1}(A^{T}+\delta I)P.$$

Since P is positive definite, this implies $A-BK_{1}+BK_{2}$ and $-A^{T}-\delta I$ are similar matrices.

Then, the following relationship holds:

(19) $$\max Re[\lambda (A-BK_{1}+BK_{2})]<-\frac{\delta }{2}.$$

Assume that γ>0 is a constant, such that $\gamma^{-2}<min\left\{Re(\lambda (R^{-1}))\right\}$, which satisfies:

(20) $$K_{1}^{T}RK_{1}>\gamma^{2}K_{2}{}^{T}RK_{2}.$$

Through some mathematical transformations, the following GARE equation can be obtained:

(21) $${\left(A+\frac{\delta I}{2}-BK_{1}+BK_{2}\right)}^{T}P+K_{1}^{T}RK1+P(A+\frac{\delta I}{2}-BK_{1}+BK_{2})-\gamma^{2}K_{2}^{T}K_{2}=0.$$

Based on the above GARE Equation (21) and the control and disturbance input policy (16) and (17), the ADP algorithm is proposed for the control of the swing phase of the exoskeleton.

ADP Algorithm for Lower Extremity Exoskeleton Control

In the controlled system, (A, B) is stabilizable, (A, $\sqrt{Q}$) is detectable, and (19) and (20) hold. Starting from an initial control, we perform the following two-step iterative process, until a given convergence accuracy $\left\|p^{k+1}-p^{k}\right\|\leq \epsilon$ is reached, where ϵ is a very small positive constant.

Policy evaluation: The kernel matrix $P^{k+1}$ is solved by:

(22) $$\begin{array}{c} 0={\left(A+\frac{\delta I}{2}-BK_{1}^{k}+BK_{2}^{k}\right)}^{T}P^{k+1}+{\left(K_{1}^{k}\right)}^{T}RK_{1}^{k}\\ +P^{k+1}(A+\frac{\delta I}{2}-BK_{1}^{k}+BK_{2}^{k})-\gamma^{-2}{\left(K_{2}^{k}\right)}^{T}K_{2}^{k} \end{array}.$$

Policy improvement: One can obtain the tracking control policy by:

(23) $$u^{k+1}=-R^{-1}B^{T}P^{k+1},$$

(24) $$\omega^{k+1}=\gamma^{-2}B^{T}P^{k+1}.$$

In the controlled system 1, (A, B) is stabilizable and (A, $\sqrt{Q}$) is detectable; if (19) and (20) hold, then the control strategy 23 can make the controlled system stable, and for the detailed proof, one can refer to [37,38,39].

This is a value iteration (VI) ADP structure. The VI algorithm needs to be able to fully learn all states of the lower extremity exoskeleton system. The detection noise can be used to meet the persistent excitation conditions, and a longer training time is required.

When using the ADP algorithm to solve the tracking control, it is necessary to introduce a noise signal to satisfy the persistent excitation condition, so that the controller can traverse all states of the system to achieve a good learning effect.

## 6. Simulation

In this section, the ADP algorithm is used as the control policy of the lower extremity exoskeleton to improve the angle tracking accuracy of the exoskeleton. The performance index function was designed in (14), where R and Q were selected to be unit matrices with the appropriate dimensions.

According to the dynamic model of the exoskeleton, the parameters required in the simulation include the mass of the pole, the rotational inertia of the pole, the length of the rod, and the distance from the center of the mass of the pole to the rotational joint. The parameters of the exoskeleton are shown in Table 2.

**Figure 10:**
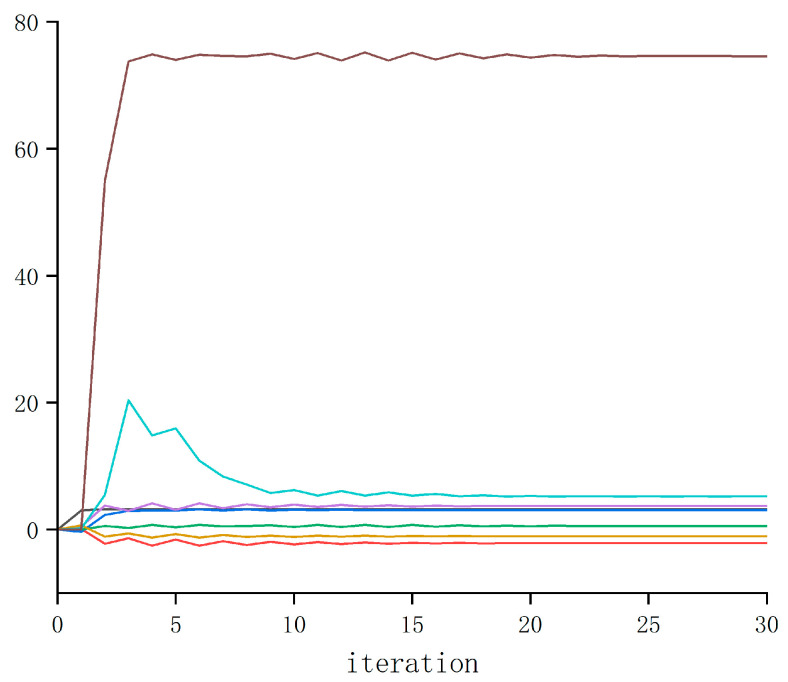
The training progress of P.

The iterative learning process of the ADP algorithm is shown in Figure 10. Through sufficient study of the lower extremity exoskeleton system, the converged P matrix of policy evaluation (22) was obtained.

**Figure 11:**
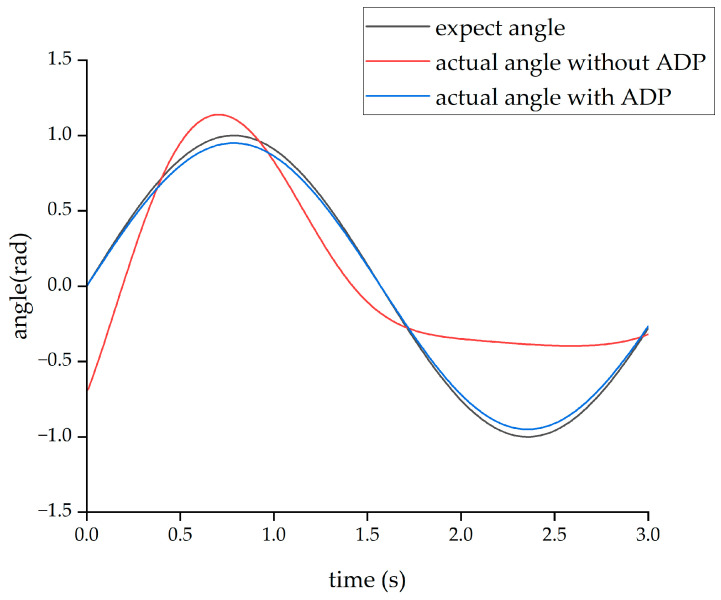
Tracking effect of the hip joint with sinusoid input.

**Figure 12:**
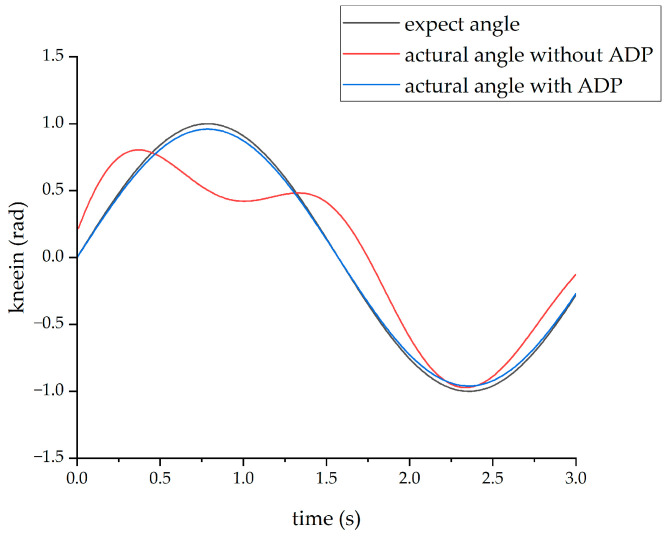
Tracking effect of the knee joint with sinusoid input.

After completing the learning process of the ADP algorithm, two forms of input signals were applied to evaluate and verify the effectiveness of the ADP algorithm for the control tracking of the lower limb exoskeleton. The first form of the input signal was a sine signal with a period of three seconds and amplitude of 1 radian. Under the action of this sine signal, the output angle curves of the hip and knee joints are shown in Figure 11 and Figure 12, respectively.

**Figure 13:**
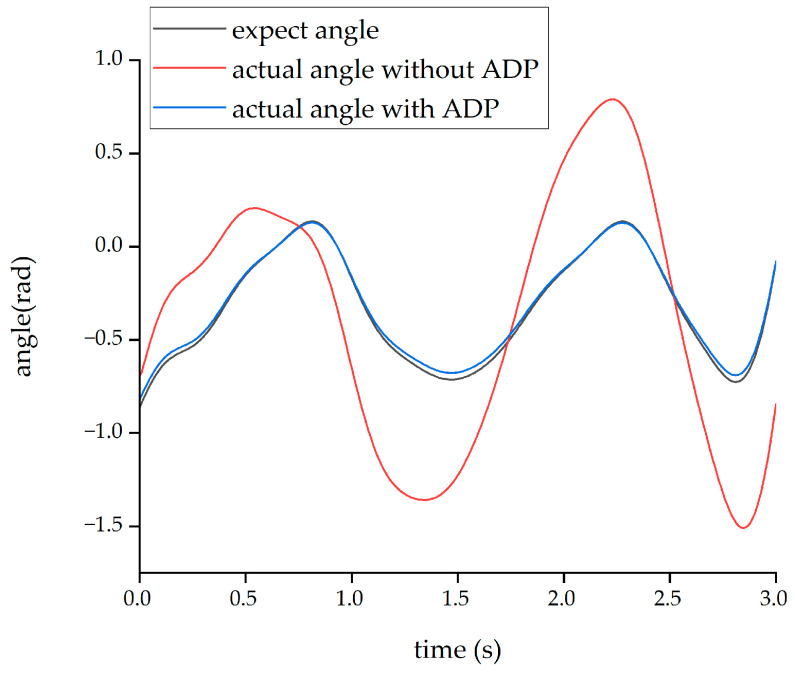
Tracking effect of the hip joint with CGA input.

**Figure 14:**
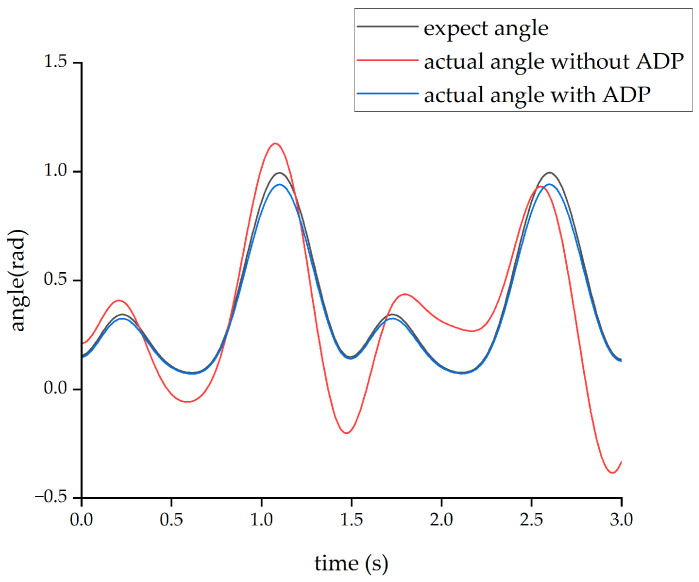
Tracking effect of the knee joint with CGA input.

The second form of input signal was obtained from the Clinical Gate Analysis (CGA) [38]. Under the action of this CGA signal, the output angle curves of the hip and knee joints are shown in Figure 13 and Figure 14, respectively.

From the above four figures, it can be seen that for both the hip joint and knee joint, the origin output angle deviated greatly from the desired output angle and could not be tracked well. However, after the ADP algorithm was adopted, the angle tracking effect was significantly improved, and the ADP output angle curve almost coincided with the desired curve. Therefore, the ADP control algorithm is an effective tracking control for the lower extremity exoskeleton.

## 7. Experiment

### 7.1. Experiment Device

**Figure 15:**
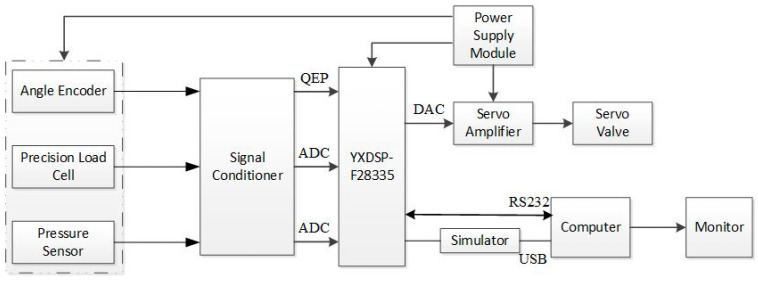
Experiment protocol of the exoskeleton.

According to the simulation results, the ADP algorithm can effectively help the exoskeleton on position tracking, but in practical systems, the position tracking process between the exoskeletons and pilots is also affected by wear matching, friction, and other factors. Therefore, experiments on the exoskeleton platform are described in this section. The control diagram of the system is shown in Figure 15.

**Figure 16:**
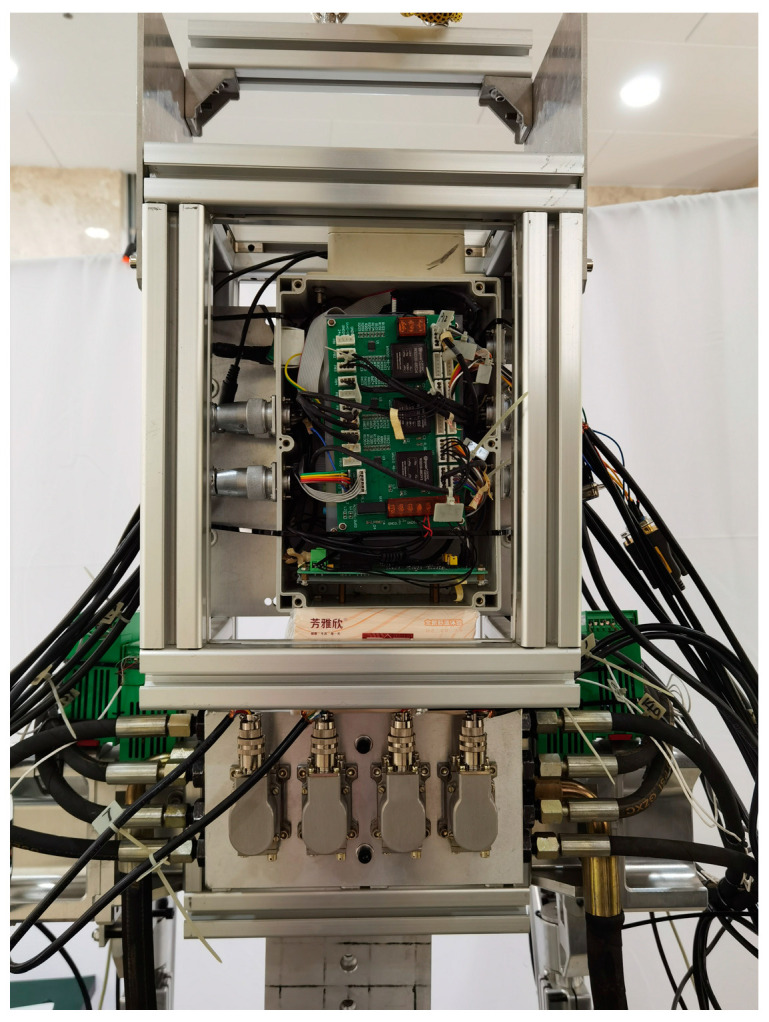
Installation structure of the measurement and control system.

The system included a main signal processor, simulator, sensor, signal conditioner, servo amplifier, and computer host. The DSP adopted the YXDSP-F28335 Ultimate Edition development board from Yanxu Company, which uses the TMS320F28335 digital signal processor (DSP) developed by TI Company. The DSP was connected to a computer through an emulator for programming and debugging. The sensor was connected to the DSP input port through signal conditioning, using differential connection. Twelve channels of ADC collected four hydraulic cylinder tension pressure sensor signals and two plantar pressure sensor signals, and four channels of the QEP collected four joint encoder signals. Four servo amplifiers were connected to the DSP 4-channel DAC output terminals to control the input current of the servo valve and achieve hydraulic cylinder output force or displacement control. The power module provided the required DC power supply for the sensors, amplifiers, etc. The hardware of the measurement and control system was installed behind the exoskeleton back frame, which is shown in Figure 16.

**Figure 17:**
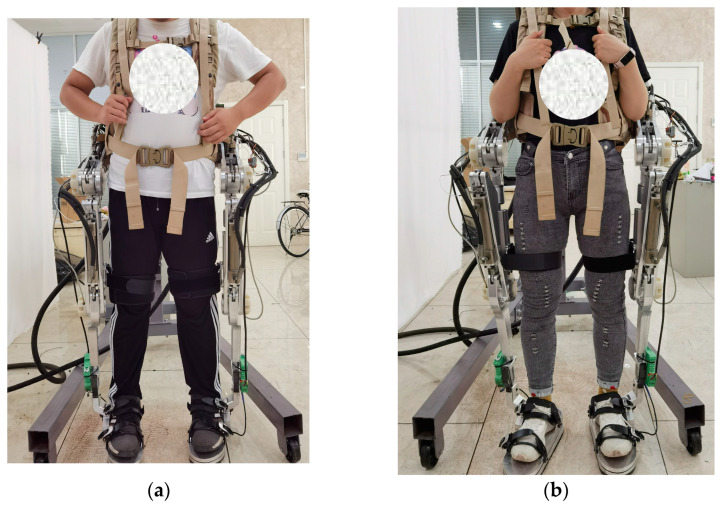
Pilots during the experiment. (**a**) Pilot No. 1. (**b**) Pilot No. 2.

In order to evaluate the performance of the ADP control algorithm in exoskeleton tracking control, joint angle tracking experiments of the lower limb exoskeleton robots were conducted on the experimental platform shown in Figure 17. Since the control process only considers the joint angle and torque of the human in the forward direction, in order to prevent the degrees of freedom in other directions of the exoskeleton structure from affecting the experimental results, the non-driving degrees of freedom of the hip and ankle joints were limited, so that during the experiment progress, the pilot could only walk straight forward.

Two pilots participated in this experiment. Pilot No. 1 was 30 years old, weighed 80 kg, and was 1.80 m tall, and pilot No. 2 was 26 years old, weighed 60 kg, and was 1.65 m tall. Each pilot corresponded to the longest and the shortest state of the thigh and the shank.

During this experiment, the pilots wore protective gear. Prior to the start of the experiment, the pilots adapted to the exoskeleton system and were familiar with the operation of the exoskeleton. The hydraulic pump station used in this experiment had an emergency stop button that could unload the system with one click.

### 7.2. Experiment Results

As the model and algorithm process in this work took the swing phase as an example, the experimental process began with the pilots wearing the exoskeleton, which was unactuated; the pilots were supported by one leg and performed periodic swings on the other leg. The position information was collected by the angle encoders installed on the joints, which served as the expected signals for the exoskeleton system.

Afterwards, the driving component of the exoskeleton, namely the hydraulic cylinder, was installed in the corresponding position, and the hydraulic cylinder was used to drive the exoskeleton. The position information collected by the angle encoder at this time was the actual position tracking signal of the exoskeleton.

The difference between the expected position of the exoskeleton and the actual position output of the exoskeleton was the control error of the exoskeleton.

**Figure 18:**
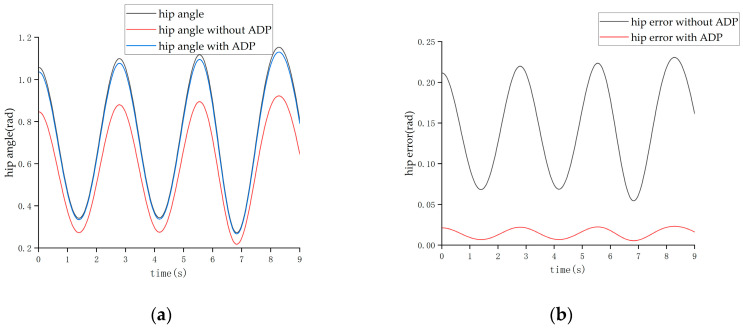
Tracking effect of the hip joint in the walking state. (**a**) Angle of the hip joint. (**b**) Tracking error of the hip joint.

**Figure 19:**
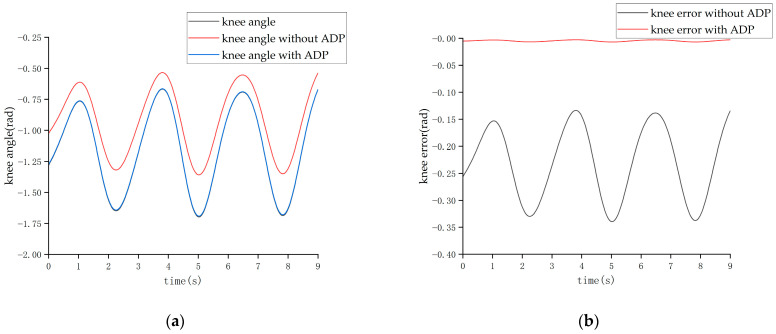
Tracking effect of the knee joint in the walking state. (**a**) Angle of the knee joint. (**b**) Tracking error of the knee joint.

**Figure 20:**
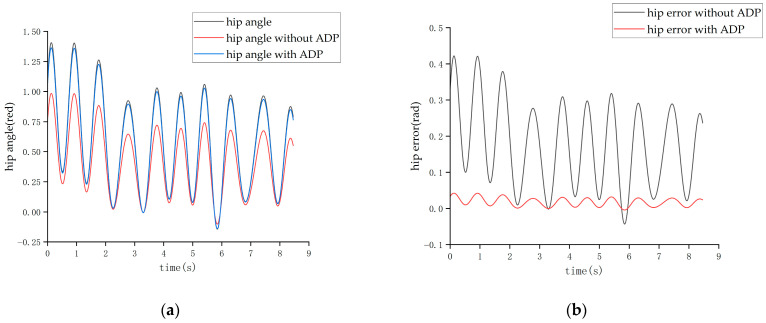
The tracking effect of the hip joint in running state. (**a**) Angle of the hip joint. (**b**) Tracking error of the hip joint.

**Figure 21:**
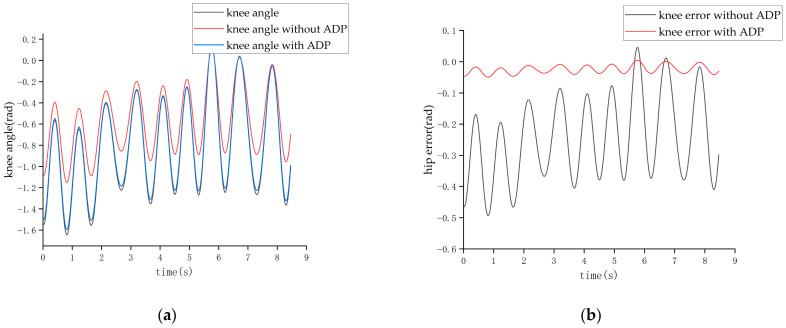
Tracking effect of the knee joint in the running state. (**a**) Angle of the knee joint. (**b**) Tracking error of the knee joint.

Due to the normal walking cycle of the human body being about 3 s (1.5 s per step) and the running cycle being about 1 s (0.5 s per step), during the experiments, two sets of position information were collected when the exoskeleton was non-actuated, which were the slow-swing state and the fast-swing state. The two sets of swing states simulated the walking and running processes of the pilot. Then, two sets of experiment were conducted. In the first experiment, the ADP algorithm was not added in the control loop; while in the second one, the ADP algorithm was added. These two sets of experiments were carried out under the condition that other system parameters, including the parameters of the hydraulic control system in the lower level, remained unchanged. The experimental result of pilot No. 1 is shown in Figure 18, Figure 19, Figure 20 and Figure 21.

The tracking effect of the walking state is shown in Figure 18 and Figure 19.

It can be seen from Figure 18 and Figure 19 that when the cycle of the swing was roughly 3 s, the maximum error of the hip joint when the ADP algorithm was not added was 0.22 rad, and the maximum error of the knee joint was −0.32 rad, which are both approximately 20% of the maximum angle. After adding the ADP algorithm, the error was reduced to approximately 2%.

The tracking effect of the running state is shown in Figure 20 and Figure 21.

From the above two figures, it can be seen that when the cycle of the swing was roughly 1 s, the set of experiments without the ADP algorithm had a maximum hip joint angle tracking error of about 0.4 rad and a maximum hip joint angle tracking error of about—0.5 rad, reaching a tracking error of about 21%. After adding the ADP algorithm, the joint angle error was reduced to approximately 3%.

**Figure 22:**
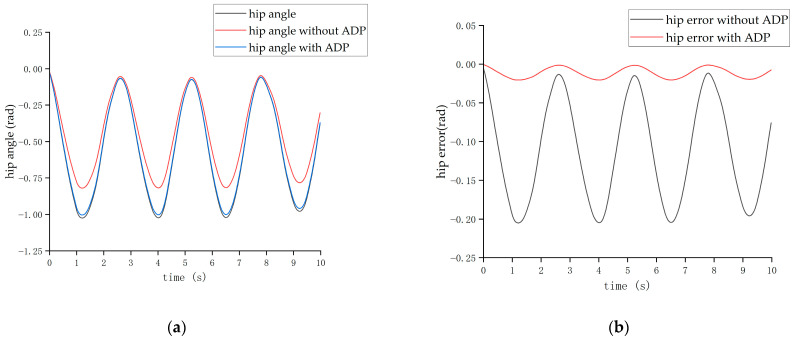
Tracking effect of the hip joint.. (**a**) Angle of the hip joint. (**b**) Tracking error of the hip joint.

**Figure 23:**
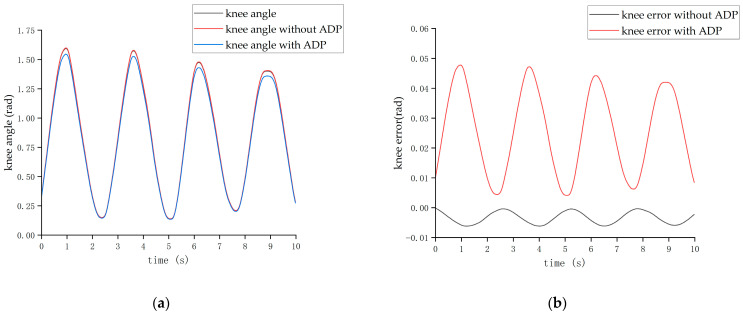
Tracking effect of the knee joint. (**a**) Angle of the knee joint. (**b**) Tracking error of the knee joint.

It was found that the height of the pilot and the length of each part of the exoskeleton did not affect the optimization effect of the ADP algorithm on tracking. Therefore, for the experimental results of pilot No. 2, only the experimental results of the walking state are shown in Figure 22 and Figure 23.

The experiments showed that when the ADP algorithm was not added into the control loop, the tracking effect was poor, introducing a significant burden to the operator. After adding the ADP algorithm, the joint angle error curve was remarkably reduced, and the exoskeleton evidently improved the joint angle tracking effect of the wearer, resulting in a significant increase in the comfort of the pilot.

## 8. Conclusions

In this paper, the design of a new type of lower limb exoskeleton with 12 degrees of freedom was presented. Taking the motion of the hip and knee joints in the sagittal plane as the research object, the dynamic model of the lower limb exoskeleton was established. A model-free ADP algorithm was designed to realize the tracking control of the exoskeleton. The proposed ADP structure can guarantee the solving accuracy and avoid the dimension disaster problem, thus effectively improving the control accuracy and calculation efficiency. The ADP algorithm greatly reduces the error of the angle tracking caused by inaccurate dynamic model, system nonlinearity, and external disturbances. The ADP control algorithm lays a good foundation for the lower level control and provides an effective control method for the high-precision control of the exoskeleton.

## Figures and Tables

**Table 1:** Design specifications of the exoskeleton.

	Min	Max
thigh length (m)	0.340	0.371
shank length (m)	0.363	0.396
hip angle (°)	−10	90
knee angle (°)	0	120

**Table 2:** Parameters of the exoskeleton.

Item	Parameter	Item	Parameter
$m_{t}\;(\mathrm{kg})$	3.57	$m_{s}\;(\mathrm{kg})$	3.97
$I_{t}\;(\mathrm{kg}\cdot m^{2})$	0.82	$I_{s}\;(\mathrm{kg}\cdot m^{2})$	1.41
$L_{t}\;(m)$	0.423	$L_{s}\;(m)$	0.466
$L_{Gt}\;(m)$	0.261	$L_{Gs}\;(m)$	0.133

## Data Availability

The data used to support the findings of this study are included in the article.
